# Coupling Adsorption-Photocatalytic Degradation of Methylene Blue and Maxilon Red

**DOI:** 10.1007/s10895-022-02934-1

**Published:** 2022-04-06

**Authors:** Rania Farouq

**Affiliations:** Petrochemical Engineering Department, Pharos University in Alexandria, Alexandria, Egypt

**Keywords:** Methylene blue, Maxilon red, Coupling adsorption-photodegradation, Rice straw, TiO_2_

## Abstract

The MB and MR removal process by two mechanisms of adsorption using rice straw (absence of UV light) and photodegradation on TiO_2_ surfaces was investigated. MB and MR removal efficiency were further intensified upon the sequential operation of adsorption followed by photocatalytic degradation over TiO_2_ under visible light irradiation. The TiO_2_ was used to remove methylene blue (MB) and Maxilon Red (MR) dye from aqueous media by continuous mode at 25 ± 2 °C, at pH 6.8 ± 0.2. Photo-illumination study revealed 75.81 and 65.51% MB and MR removal with the dose of 1 g/L TiO_2_ with an initial concentration of 5 mg/L within 120 min. This study can be deemed of potential applications for the removal of MB and MR dyes on an industrial level using the synergistic adsorption-photocatalytic oxidation approach. A probable photodegradation mechanism was proposed.

**Supplementary Information:**

The online version contains supplementary material available at 10.1007/s10895-022-02934-1.

## Introduction

Water-soluble organic dyes are discharged into rivers and sewers and thus lead to the pollution of drinking water and the environment [1–3]. Endorsed to the detrimental environmental impacts, dyes need to be removed before it enters into the water-ecosystem. To achieve this task, various approaches such as coagulation, adsorption, membrane, ion exchange, chemical oxidation, and photocatalysis have been adopted with gradual improvements over the years [4–6]. Among these techniques, adsorption has been proven to be the most competitive method owing to its low cost, simple design, easy operation, small amounts of harmful byproducts, the availability of a wide range of absorbents and easy retrieval of them [7, 8]. However, a major hindrance in such processes is a saturation of adsorbent which either requires regeneration or replacement. These aspects can concomitantly overburden process cost and post-process treatments; Photocatalysis, on the other hand, can breakdown particles that are difficult to remove entirely from treated water using traditional methods [9]. The enhanced adsorption of pollutants onto the adsorbent was closely followed by their transfer to the surface of the photocatalyst, demonstrating a full photodegradation process, according to the studies [10].

For the dye removal process, photocatalytic degradation has been endorsed close attention due to its green and efficient nature, degradation process proceeded by the dye molecule rupture that led to formation of less complex organic compounds, followed by their total mineralization [11–15]. However, many of the photocatalysts own low adsorption capacity for organic pollutants and the limited role of active species like photogenerated holes and some free radicals [16]. These shortcomings can be tackled via the integrated application of adsorption and photocatalysis for the removal of organic dyes from wastewater. The synergy strategy of adsorption and photocatalysis is a newly emerging technology in the field of water pollution control [17]. The low-concentration pollutants in water will be firstly adsorbed and enriched in the structure and then oxidized in-situ via a photocatalytic process. The adsorption followed by photo-catalysis could degrade the adsorbed organic dyes in a shorter time, therefore can considerably increase adsorbent lifetime. The above method also solves the saturation problem in the adsorption method and the separation problem in the photocatalytic degradation method [18–20].

An important aspect of the coupled application of adsorption and photocatalysis is the development of a suitable adsorbent. In this connection, the fabrication of alkalinized g-C_3_N_4_ and its composite with TiO_2_ was utilized to study the synergetic effect of adsorption and photocatalysis on the degradation of methylene blue [21]. Also synthesize natural polymer nanocomposite and employing it for coupled adsorption- photocatalytic degradation of crystal violet was investigated [22]. For the elimination of basic yellow 28 (BY28) and basic blue 41 (BB41), Boumaza et al. used activated carbon made from wild olive stones [23]. To eliminate ciprofloxacin, Song et al. made biochar from agricultural waste corn straw and combined it with BiOBr [24]. Combining photocatalyst with adsorbent would synergize the dual advantages from each component [25–27].

Rice straw was employed as a low-cost adsorbent in this work to remove the textile dyes Maxilon Red and methylene blue from aqueous solutions. Adsorption of Maxilon Red dye onto mineral adsorbents has been studied in a few types of research. The adsorption of the Maxilon Red dye on agricultural wastes has not been studied. The impact of several factors including contact time, initial adsorbent dosage, and dye concentration were investigated.

In the degradation of MB and MR, behavior, synergetic effect, and mechanism of adsorption and photocatalysis were investigated. The goal of this study was to investigate the usage of rice straw as a biosorbent and its possible application in color removal.

## Experimental

### Materials

Rice straw was washed and ultrasonically removed from the surface adherents, and then dried at 60 °C in an oven to constant weight; The dyes were obtained from commercial market and of an analytical grade. Methylene blue (MB) an organic chloride salt having 3,7-bis(dimethylamino) phenothiazin-5-ium as the counterion and Maxilon Red (MR) dye also known as Astrazon Red FBL; C.I. Basic Red 46; Cationic Red GRL, it’s chemical structure is 1,2- dimethyl-3-((4-(methyl(phenylmethyl)amino)phenyl)azo)-1,2,4 triazolium bromide, formulated into a stock solution with a mass concentration of 1 g/L, was diluted to the required concentration according to the experimental needs; experimental water is deionized water. The diameter of the TiO_2_ (anatase > 99%) crystals was between 5 and 10 nm and the specific surface area SBET was about 320 m^2^/g. All the chemicals were used as received without further purification.

### Alkaline Treatment

The alkaline treatment break the lignocellulosic structure and the lignin components are depolymerized.

When raw rice straw fibers are exposed to an alkaline solution, the hemicelluloses and lignin components contained in the raw fibers dissolve, the hemicellulose is partly hydrolyzed, and the lignin is depolymerized [28]. To improve the efficiency of the procedure, two drops of red-turkey oil were added, and the mixture was continually agitated at 170 °C for one hour.

The residual lignin on the cellulose surface was then removed by washing the mixture with 0.1 M NaOH. After filtering through nylon cloth, the carbon product was washed with acetic acid and distilled water before being dried at 60 °C for 16 h.

### Sequential Adsorption and Photocatalytic Degradation of MB and MR

#### Adsorption Experiments

Adsorption tests were conducted in the Fixed-bed column to assess dynamic behavior for the removal of MB and MR. To this end; the effects of dosage and initial concentration were pre-assessed on MB and MR removal. The adsorption kinetics of MB and MR was investigated at 25 °C. Briefly, MB and MR solutions were allowed to flow through the rice straw filter, and the samples were withdrawn at the outlet for MB and MR quantification. The adsorption results were obtained with MB and MR ranging from 5 to 15 mg /L at 25 °C.

The continuous biosorption system consisted of a feed tank, a peristaltic feed pump, a fixed-bed column with inlet and outlet ports, and a fraction collector. The column tests used a cylindrical column with an interior diameter of 4.5 cm and a height of 17 cm.

It is widely known that wall effects can affect the form of biosorption break-through curves in fixed-bed systems and that the ratio of column diameter (D_C_) to particle diameter (D_p_) should be larger than 10 to avoid such impacts. The rice straw was stacked in the column in varying amounts.

Using a peristaltic pump, the tests were carried out by flowing MB and MR solution through the biosorbent bed at a specified flow rate in an upward-flow mode. The adsorption capacity (mg/g) and efficiency (%) of selected samples at equilibrium (q_e_) and at time t (q_t_) was calculated using the following equation [29]:

1 $${q}_{t}=\frac{\left({C}_{o}-{C}_{t}\right)V}{W}$$

where C_o_ is initial concentration and C_t_ (mg/L) is the concentration at time t, V is the volume of solution (L) and W is the mass of adsorbent (g). A value of q_e_ is obtained using the same Eq. (2) with the difference of only C_e_, the concentration at equilibrium.

2 $$\mathrm{adsorption}\ efficiency\ \%=\frac{\left({C}_{o}-{C}_{e}\right)}{{C}_{o}}*100$$

#### Annular Continuous Reactor

The tubular reactor equipped with a 300 W Xe lamp (PLS-SXE300C) as simulating solar light which operates in continuous mode, is a cylindrical Pyrex reactor (ID = 5 cm, and L_TOT_ = 120 cm) because this type of geometry is the simplest configuration for a photo-reactor, allowing a possible scale-up of photocatalytic systems for water and wastewater treatment.

The stock solutions containing the MB and MR dye (at 5, 6, 9, 10,and 15 mg/L initial concentration) were prepared and collected in the feed tank (4 L). The feed solution was pumped from the feed tank to the rice straw filter. TiO_2_ is injected into the liquid stream before it is fed to the cylindrical Pyrex reactor and finally comes out from the reactor, being conveyed in a tank where the treated solutions were collected. The liquid sample was withdrawn at the outlet of the continuous flow reactor. The amount of TiO_2_ sample, used in the experimental tests, was equal to 1 g/L.

**Fig. 1:**
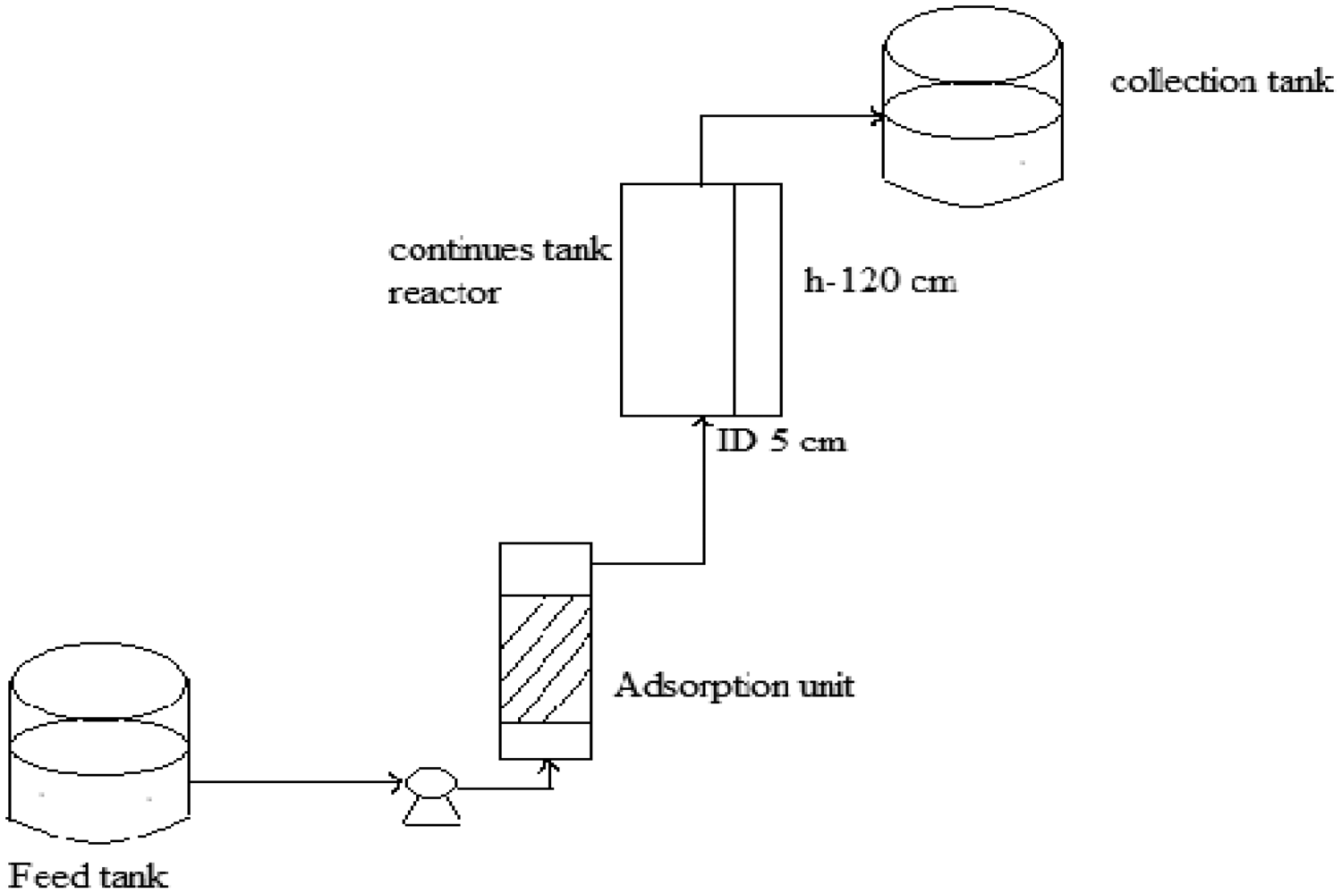
The schematic diagram of laboratory- scale of the continuous flow reactor

The schematic diagram of laboratory- scale of the continuous flow reactor is shown in Fig. 1.

## Results and Discussion

The adsorption-photocatalytic performances of rice straw- TiO_2_ were evaluated using MB and MR as model dye pollutants.

### Biosorbent Dosage

The efficiency of sorption processes is highly influenced by the quantity of biosorbent and contact time. The effect of biosorbent dosage was studied using 5, 10, 15, 20, and 25 g to remove methylene blue and Maxilon Red in batch sorption systems.

**Fig. 2:**
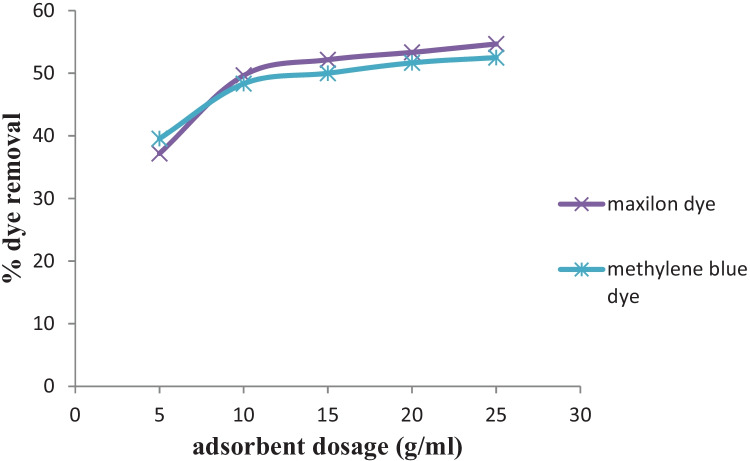
Effect of adsorbent dose on removal efficiency of MB and MR dye onto the prepared adsorbents at pH 6 (25 °C ± 2), initial dye concentration = 6 mg/L, and volume = 250 mL

Figure 2 shows that MB and MR removal increased from 39.55 and 37.18 to 50 and 52.15% with the increase of rice straw from 5 to 25 g. This behavior is explained by the increased number of sorption sites available, in this case in the higher mass of rice straw. Further increment of adsorbent does not affect much due to non-availability of the adsorbate.

### Effect of Adsorption Process only at Different Initial Concentration

The initial dye concentration has a significant impact on the dye degradation from aqueous solutions.

**Fig. 3:**
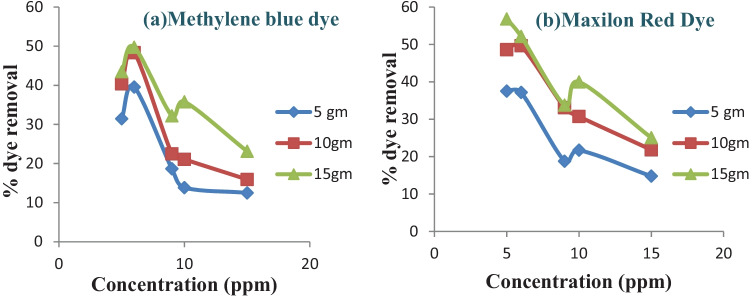
Effect of initial dye concentrations on the removal efficiency of MB (**a**) and MR (**b**) at pH 6 (25 °C ± 2) at different biosorbent doses

At room temperature, the adsorption of MB and MR on rice straw was studied at various initial dye concentrations, and the outcome is shown in Fig. 3.

Upon increasing the initial concentration from 5 to 6 ppm the rate of degradation increases due to the increase in the mass transfer from the concentration gradient. Whereas the initial dye concentration increases beyond 6 ppm the adsorption percentage drops. This is due to limited adsorption sites available for the uptaking of dye [30]. For the constant dosage of the adsorbent, at higher initial dye concentration, the available adsorption sites of adsorbent become fewer, and hence the removal of dye depends upon the initial concentration.

### Kinetics of the Process

**Fig. 4:**
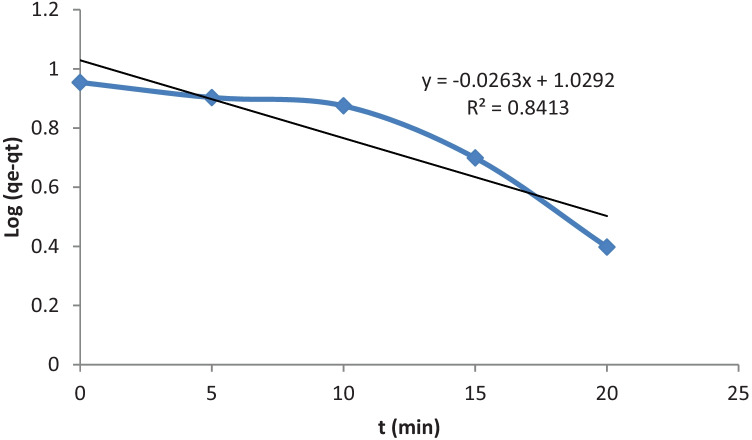
Pseudo-first-order kinetic model

Adsorption kinetics is usually governed by film diffusion and intra-particle diffusion. However, the adsorption capacity and the rate required for the equilibrium concentration were investigated using the pseudo first-order-kinetic model. Dye molecules adsorbed from the aqueous solution increased quickly over time, and the equilibrium was achieved within 60 min for the two dyes. The kinetic model shown in Fig. 4 fitted well to the experimental data with the coefficients of determination (R^2^) higher than 0.8.

The photocatalytic degradation of MR and MB in presence of TiO_2_ as catalysts was investigated. The Langmuir–Hinshelwood (L–H) model is generally used to describe the kinetics of photodegradation of organic pollutants as the reaction mainly occurs between the adsorbed substrates on the catalyst surface and the photogenerated oxidants. The L–H kinetic equation could be expressed as follows [15]:

3 $$r=\frac{{{K}_{a}k}_{r }C}{1+{K}_{a}C}$$

where r is the rate of photodegradation, C is the dye concentration at time t, k_r_ is the rate constant, and K_a_ is the adsorption equilibrium constant. The equation can be simplified to an apparent first-order equation:

4 $$\mathrm{ln}\left(\frac{{C}_{o}}{C}\right)= {K}_{a}{k}_{r}\ t={k}_{o}\mathrm{t}$$

**Fig. 5:**
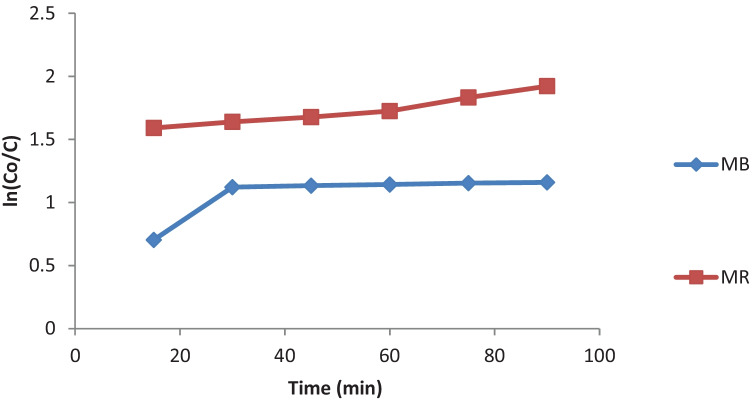
Pseudo-first-order kinetic plot for the degradation of MB and MR

A plot of ln (C_o_/C) vs. time is represented in Fig. 5.

The values of k_o_ and the linear regression coefficients of photodegradation of the red dye which correspond to different dyes are listed in Table 1.

**Table 1:** Pseudo-first order apparent constant values for the different dyes

**Dye**	**k**_**o**_** (min**^**−1**^**)**	**R**^**2**^
MB	0.0051	0.7464
MR	0.0047	0.9753

The kinetic and regression constants are presented in Table 1. The reaction rate constants (k_app_) were 0.0051and 0.0047 min^−1^for the MB and MR, respectively.

### Effect of Photocatalytic Illumination at Different Initial Dye Concentrations

**Fig. 6:**
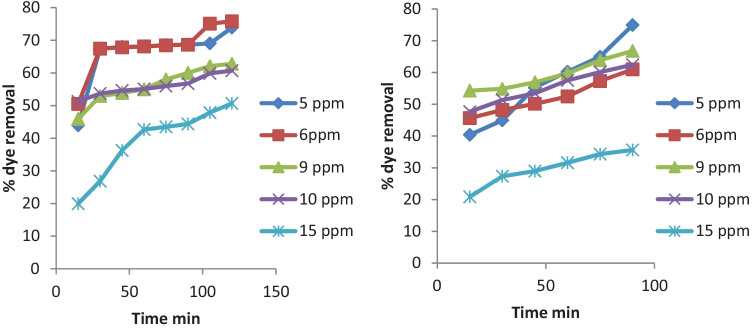
Effect of initial dye concentration on the photodegradation by TiO_2_ (**a**) MB dye (**b**) MR dye

The initial concentration of organic pollutants in contaminated water is a significant parameter that affects the efficiency of the treatment process. The dye concentration diminished with time throughout the treatment procedure, as seen in the time-dependent concentration profile of dye during photodegradation (Fig. 6). This implies that as the photocatalytic process continued, the dye molecules were destroyed. The results also reveal that the degradation efficiency of TiO_2_ reduced as the starting dye concentration was raised from 5 to 15 mg/L. This suggests that the system's hydroxyl radical production is insufficient for efficient dye degradation at greater concentrations [31]. According to the Beer-Lambert equation, when the initial dye concentration rises, the path length of photons crossing the solution shortens, resulting in decreased photon absorption on catalyst particles and lower photocatalytic reaction rates.

Furthermore, when the concentration of the substance grows, the solution gets more turbid, limiting the number of photons that reach the catalyst surface. So, the amount of hydroxyl radicals attacking organic molecules is limited, lowering the degradation efficiency.

### Adsorption and Photocatalysis Performance and Coupling Effect

**Fig. 7:**
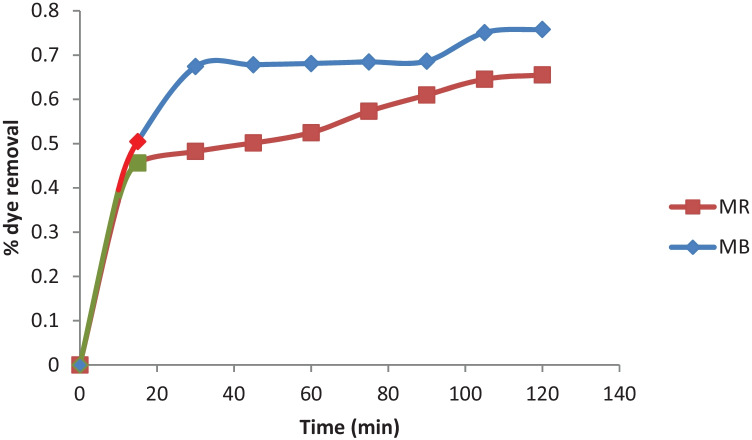
Coupling effect of Adsorption and photocatalytic performance of MB and MR

Rice straw exhibited a remarkable adsorption capacity for MB and MR, and the removal efficiency reached 39.55 and 37.18% for MB and MR after adsorption. However, MB could not be fully removed. To increase the efficiency of removal, the photocatalytic process was preceded under the irradiation of visible light. The data compiled in Fig. 7 demonstrate that MB and MR could reach 75.81 and 65.51 after 120 min under the combined effect of adsorption over rice straw followed by irradiation under visible light following the coupling mechanism of the proposed adsorption-photocatalytic process.

### Comparison of Degradation of the Dyes

The effect of molecular structure and functional groups on photocatalytic degradation was studied by various researchers [32, 33]. Methylene Blue and Maxilon Red differ in their molecular structure, and functional group, as well as in the extent of ionization in an aqueous solution.

Both the dyes were treated at the same experimental conditions, viz. catalyst dose = 1 g, dye concentration = 5, 10 and 15 mg/L, and pH = 7.0. The photocatalytic degradation extent after 2 h was determined by the absorbance reduction of the solution. It was 75.81% for Methylene Blue, compared to 65.51% for Maxilon Red.

As adsorption is a prerequisite for photocatalysis, this may be due to the higher extent of adsorption in the case of Methylene Blue than in the case of Maxilon Red this may be because of the existence of only one hydroxyl group as a substitute; and to the fact that the resonance effect of a substituent works only when the hydroxyl group is directly linked to the unsaturated system. As a result, to describe the influence of the hydroxyl group on the reactivity of the organic material, only the field effect (−1) must be considered. The hydroxyl group's amount in the dye molecule can boost this resonance and likewise, the degradation rate of the dye [34].

### SEM Studies

**Fig. 8:**
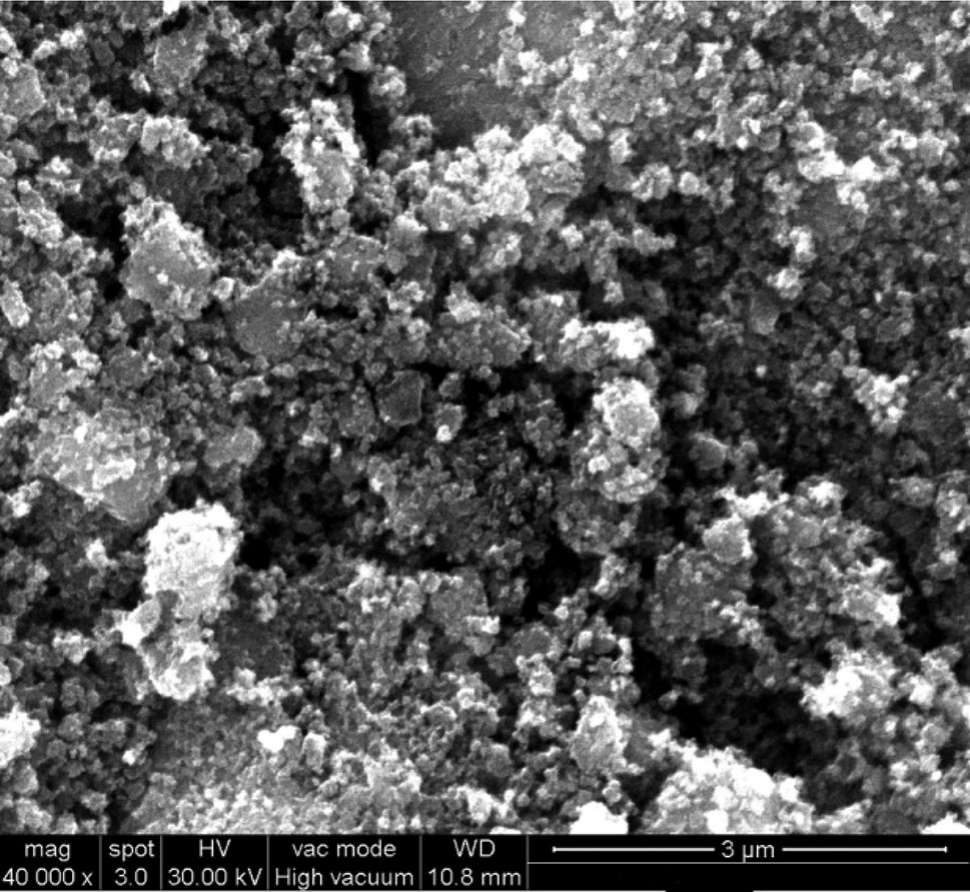
SEM image of TiO_2_

Scanning electron microscope (SEM) images of TiO_2_ is shown in Fig. 8. It clearly shows very closely packed spherical and bubbles shaped nanocrystals.

## Conclusions

The degradation of Methylene Blue and Maxilon Red dyes using the coupling adsorption-photocatalytic oxidation approach was proven to be effective in the removal of these dyes from an aqueous solution.

The degradation efficiency has been generally, found to increase with the increase in catalyst loading, and a decrease in initial concentration. Of the two dyes, Methylene Blue was degraded faster.

The rate equation for the photocatalytic degradation process followed pseudo-first-order kinetics and the rate-constants were determined, using Langmuir–Hinshelwood model. The kinetic model was based on hydroxyl radical attack. Therefore, this simple technology of photocatalytic degradation of the colored effluents has the potential to improve the quality of the wastewater from textile and other industries. The economy may be further improved using certain modifications.

## Supplementary Information

Below is the link to the electronic supplementary material.

Supplementary file1 (XLSX 44 KB)

## Data Availability

Not applied.

## Code Availability

Not applied.
